# Central Dental Association of Northern New Jersey

**Published:** 1896-09

**Authors:** 


### CENTRAL DENTAL ASSOCIATION OF NORTHERN NEW JERSEY.

A regular meeting of the Association was held on Monday evening, May 18, 1896, at 943 Broad Street, Newark, the President, Dr. Walter Woolsey, in the chair.

*Dr. Sutphen.*—I would call the attention of the members to the proceedings of the annual meeting, which have been printed and are here for distribution.

*Dr. Meeker.*—We have on the table here this evening some copies of the "Dictionary of New Remedies," a very valuable thing for the dentist, which has been presented by Dr. Adams with the compliments of McKesson & Robbins.

*Dr. Barlow.*—Mr. President, the Executive Committee have to report that Dr. Barrett, who, as you know, disappointed us at the last meeting, will give us a lecture either in September or October. He has new slides prepared expressly for this exhibition, and Mr. Fiske, of New York, will provide the lights and show the slides.

*Dr. Meeker.*—Mr. President, I do not wish to interfere in any way with the progress of the meeting or the paper of the evening by Dr. Twilley, but he will excuse me if I speak for a minute of these subjects, because this is the last meeting until September. The New Jersey State Society meeting commences at Asbury Park on July 15. We have already received assurances of attendance from various sections throughout the country, and we think that this will be the largest exhibition the Society has had, even larger than that we had last year.

*The President.*—The next thing in order is the paper of the evening, by Dr. William Shaw Twilley, of Baltimore, entitled, "Dentistry, Practical and Theoretical."

(For Dr. Twilley's paper, see page 563.)

*The President.*—Gentlemen, the paper is now open for discussion. I will call upon Dr. Eaton to speak on this subject.

*Dr. Eaton.*—Mr. President, I do not know that I have anything of merit to advance in regard to the paper. I have had a theory for a long time—it is simply a theory—that the dentists of to-day should be the judges of the qualifications of the men who are to practise dentistry hereafter,—that is, that every man who anticipates studying dentistry should be required to serve some time in the office of a preceptor; and that preceptor, if he is an honest man, finding that a young man is not adapted to the practice of dentistry, would tell him so, kindly and candidly, and advise him to seek some other means of gaining a livelihood. To make a dentist, a man must have some special endowments; in addition to a good and active brain he must have a natural love of mechanics if he expects to succeed in dentistry; and that is the point I want to make, that his preceptor should be the judge of those qualifications.

*Dr. J. Allen Osmun.*—Mr. President, this is a subject in which I

have taken a great deal of interest, and I think some of you have heard my views on it before. I do not think I have the faculty of presenting the question quite so lucidly and gracefully as Dr. Twilley has done to-night; nevertheless, I endorse all his propositions heartily; and I am glad to see, from Dr. Eaton's suggested method of procedure, that he believes in probation. I believe that every young man who enters the dental ranks should be required to serve a time on trial. If he is found to be qualified, if he possesses the natural abilities, then promote him; otherwise turn him off in some other direction. I have claimed before, and all the succeeding years of experience have not changed my opinion, that you cannot make a dentist. Dentists cannot be made to order; they are born; the qualifications are given by the Master Workman, and the colleges, the private preceptors, and the examining boards simply train them to further usefulness in larger fields. I was glad to hear Dr. Twilley say that practice without theory is like faith without works,—dead. I believe the theoretical man is a weak man, and I regard the practical man without theory as equally weak. It is the union of the two, the theoretical and the practical, that makes the complete man. It is a mistake to develop one side of a man's nature at the expense of the other side.

I am glad this question has come up. I think that if the colleges are to be criticised at all they can be criticised on that particular side,—that they are elevating theory at the expense of practice. A college student having great ability as a mechanic, who does plate-work, and makes beautiful crowns, is unable to pass the examination in the theoretical branches. Is that right? Theory is necessary to a certain degree, but theory will not do the work; theory does not preserve the teeth; it will not place the gold of a filling in such juxtaposition to the dentine as to exclude moisture and prevent further decay. A man must have mechanical ability back of his theory. Theory may be the key-stone of the arch, but the key of the arch without the other parts is very weak. We must be symmetrical. I think the only criticism we can make against the colleges is that they are elevating theory at the expense of practice. Dr. Barlow, some years ago, expressed the opinion that students should have a larger degree of mechanical training than they were getting, and I agree with him. I think the students coming from the colleges should know how to make a set of teeth, how to swage plates, how to do creditably the various mechanical work that comes to the dentist, as well as to be filled with the pathology, anatomy, and the other things that they study, which I

doubt sometimes will be really of much value to them in every-day routine life. Yet I do not wish to be quoted here as being in the position of disparaging theory in the slightest degree. Theory and practice should go hand in hand.

*Dr. G. Carleton Brown.*—Mr. President, before I speak on this question I want to congratulate the society on the essay which they have had the pleasure of listening to to-night. It strikes the key-note to the advance of dentistry. I am delighted to have heard it from the source it comes from, a representative of the colleges. It shows the plane on which the colleges stand. The examining boards are criticised and the colleges are criticised; they both probably make mistakes, but it is not from intention.

Dr. Eaton spoke of the necessity of young men being put on probation with a preceptor. Now, that theory is a good one if it could only be carried out; but how many of the young men who go to college study first with a perceptor? Not one out of a hundred. That being the case, we have to call on the colleges to act in the place of the preceptor. I think I brought that question up a few months ago before the society, with reference to the statement of Dr. Crouse in his journal, that the colleges should require of a man before he was finally started in his course a certain amount of mechanical adaptability. If the profession at large cannot find this out, why cannot the colleges take it upon themselves; and if they find a young man who has positively no chance of ever becoming a dentist, tell him so, and send him to farming or something else. As Dr. Twilley said, there is too much of the money-making idea in dentistry. The great object of the student is to get through college as quickly as possible and then get to work making money; and the men who come from the farms and the backwoods are the very men who go into the "dental associations" that have been mentioned. They go to college originally with the idea of going back to their small town and doing what dentistry there is to do; but they do not go back there. They are in the city, they get imbued with the idea of city life and quick returns, which they know they cannot get in their own village, where they might do good work; and the result is that they gravitate into some one of these dental associations that are looking for men of that stamp; they offer what appears to these young men munificent sums to come into their employ and take charge of a lot of so-called students, and they become a curse to the profession. If the colleges can take hold and help the profession at large in this matter it will be a great advantage.

Dr. Osmun, I am surprised to find, has been able to grasp an idea that I did not know had gone beyond the examining boards, that really the colleges are leaning too much to theory and too little to practice. We are finding in our examinations, almost without exception, that while the men were very much better informed theoretically than formerly, their practical work is retrograding, and not only in the mechanical but also in the operative department. We had before us in April a set of students who were remarkable in some respects, but some of those young men who passed superior examinations theoretically knew very little about practical work, and were not fitted to carry on an office of their own. I hope the colleges will come to a realizing sense of the fact that in their noble efforts to advance the profession they have run in one direction, the theoretical, to the sacrifice of another and equally important branch, the practical.

*Dr. Meeker.*—Mr. President, we are all very much pleased to have had Dr. Twilley with us at this time to read a paper, especially as he is the representative of the oldest dental college in the United States. It seems to me that the whole tenor and lesson of the essay is the one important fact that we must be up-to-date dentists. That is what we must be, and to do it we must read the periodicals, know what is going on in the dental world, and be advanced in everything. The subject of cataphoresis is now coming into prominence, and I think dentists should know all about that subject. We hear of the dental associations that Dr. Brown mentions starting up all over the country, associations that extract teeth by so-called "vitalized air" and gas for twenty-five cents. They are working to get the patronage of the people, and we must be active in antagonizing them.

*The President.*—Dr. Gibson, of New York, is with us this evening; we would be pleased to hear from him.

*Dr. Gibson.*—Mr. President, I thank you for the honor, but I have been here as a listener to the paper and to get instruction, not thinking I would be called on to take part in the discussion. The discussion led me to thinking and to wondering, if we are to have students on probation for two or three years, then require them to spend three or four years in a dental college, and then to wait from one to six years before they can pass the State board, whether a man would not be too old to practise dentistry by the time he is permitted to enter the profession; and I could not help wondering where some of us would have been if those requirements were made about the time we entered the profession. As for myself, in all probability, I would have been somewhere in the hills of Onondaga County raising potatoes. It has been my privilege and pleasure during the past ten years to give practical lectures at the Baltimore Dental College; and let me say, gentlemen, that there is a great demand on the students in that institution for a practical knowledge of dentistry. It is surprising what a man has to meet, what is expected of him before he can go out to practise; and I think that the State Board of Examiners are making a very serious mistake when they allow men to pass without a thorough practical examination. I think it is the fault of the boards of examiners that they are running altogether too much to theoretical examinations.

*Dr. Barlow.*—Mr. President, I have been very much interested in Dr. Twilley's paper, and also in the remarks of Dr. Osmun and Dr. Gibson. In my opinion dentistry is mechanical to a very great extent, even as regards the operative and surgical branches; a man who practises it must have the mechanical ability to handle tools. As regards the probationship of students, the examining boards find that those students who stand the highest and pass the best examinations are the ones who have first been under the preceptorship of competent dentists and good practical workmen, and then have gone through college. Referring to Dr. Gibson's remarks in regard to the requirements of the boards, I would like to state that the New Jersey State Board especially requires that men shall be well up in the practical branches; their percentage depends to a very large extent upon their operative dentistry and their mechanical work.

*Dr. Fish.*—I am surprised at the lack of mechanical knowledge exhibited by some of the students who come from the colleges. We find almost invariably that the colleges have gone into theory to the detriment of the mechanical and practical branches. I was astonished recently in looking over the examination papers of a young student who is in attendance at one of the colleges not many hundreds of miles from this city, to the east of us, to see that his examination was almost entirely in theory. He has been in the institution one year, and I must say that he has not learned the first rudiments of the mechanical branches of dentistry. It is all right to say that dentistry is a specialty of medicine, but you cannot escape from the fact that it is really a higher order of mechanics.

It is sad to see that the young men turned out from the colleges are almost wholly ignorant of the laws of leverage that govern operations in the mouth, in the construction of bridges and crowns and fillings, all of which bear on the laws of leverage, velocity, and resistance, which they should know. It would be better for the colleges to teach a little natural philosophy instead of giving so much time to the theoretical branches. I am heartily in accord with the work of the examining boards, and I hope the colleges will give more attention to the mechanical branches of dentistry than they seem to be doing.

*Dr. Baker.*—Mr. President, I know that the colleges are not perfect, but I do not think they are altogether to blame for the deficiencies that have been spoken of. I am positive that, with the colleges we have to-day, if a man does not know dentistry when he goes out from them it is to a great extent his own fault. The colleges have competent teachers, they are provided with all the different appliances required in dentistry, and I think the student, if he chooses to apply himself, may learn the practical part of dentistry as well as the theoretical, although there are a great many chances, as I have myself seen, for students to slight their work through certain manipulations. Of course, the practical part of dentistry is not pleasant to a great many. I have seen men come from college who, I am quite positive, had not performed their work as they should have done it; but it is not really the fault of the professors. They have men under them who are supposed to teach the students what they should know. Men do get through without acquiring practical knowledge, as you have already seen, but I think it is more the fault of the students themselves than it is of the college.

*Dr. Hull.*—I may say, Mr. President, that I think too much is expected of students. Here is a gentleman from one of the colleges who can place in a superior gold filling, can make a perfectly-fitting plate or a beautiful piece of bridge-work, and yet the college will not pass him, claiming he is not up in theory. As far as I know dentistry, it is an utter impossibility to do those things without being to a certain extent a theoretical man. It is right that a dentist should be educated, and I think they generally are fairly educated.

One thing I want to say of the State boards: I think they demand altogether too much of the student who has just graduated. They should not expect a young man to come out of college a thoroughly equipped dentist. He cannot get practice in college. He acquires theory, he gets an excellent foundation, and it rests with himself to make himself what he is to be.

*Dr. Chase.*—Mr. President, in the remarks we have had on this paper I do not think there has been anything said against it, and I shall say nothing against it. I am heartily in favor of its tenor. I think practical dentistry and theoretical dentistry must go hand in hand. A man may be able to put in a superior gold filling and to make good plate-work, and yet be absolutely unfit to treat an ulcerated tooth or prescribe the remedies that should be given in such cases, so it is necessary that he should have some theory. I believe, with the essayist, that theory and practice should go together.

*Dr. Gregory.*—When I entered college, Mr. President, I was given to understand at the outset that the college did not propose to make me a finished product, but rather to help me to gain such information as would start me out on my career,—in other words, that I was to be equipped sufficiently well that I might do my work intelligently, but not perfectly. The figure of the arch has been used here to-night as an illustration. There is one essential feature to be taken into consideration in constructing an arch, and that is the lateral thrust. I believe that if we dentists intend to practise our profession on a high plane we should educate ourselves up to that standard,—in other words, that we should have theory,—and I believe that is what the college should lay greater stress upon. Our profession is unique in that most of the men entering it have been handicapped by lack of education. Too many men seek to enter the dental profession without having had a good English education. They go to college, and the terms used there are a completely foreign language to them, they have to go to the dictionaries and lexicons in order to interpret the lectures. The only education they have when they begin to practise is what they have derived at college. If they are to be doctors or dentists or teachers they should have a broader knowledge. So I believe the colleges are right in the main, and that a man who goes through the college, if he is diligent and intelligent, will gain the information which he is afterwards to apply; and being well informed theoretically, he will, if he be honest with himself, soon find avenues that will lead to success for himself and honor to the profession he has chosen.

*Dr. Brown.*—Mr. President, I do not like to come up the second time, but I cannot resist taking advantage of the opportunity to show how opinions differ. We have had the opinions of two gentlemen from New York; one of them says the boards do not require a sufficiently advanced standard in mechanics, and the other one says the boards demand too much. Now, that simply shows that our brothers from New York do not know what is going on in New Jersey, because they are both wrong. Dr. Barlow explained to a certain extent the requirements of the New Jersey Board in the direction of mechanical skill, and I would like to supplement his statement by saying that the men who come before our board have to show their proficiency in the mechanical branches before their theory is considered at all. A man who is not properly up in practice cannot get through the board, no matter how much theory he has. The other gentleman says the boards require too much, and that the colleges require a preliminary education to a certain standard; and he is wrong in both cases. The colleges, as a rule, do not, I believe, require any preliminary education at all. They say they do, but if the men who come out of the colleges ever had any preliminary education they have forgotten it before they get through, as their performances before the boards show. Such exhibitions of English you never heard in your lives as some of the graduates of the colleges present. In the *Western Dental Journal* of two months since, I think, I was called down for my paper that I read before this Association on this very subject, and it was said in the article referred to, written by the editor, who is a college professor, that some one was playing a huge joke on the Association, that some one, who had never been inside of a dental college, had come before the Examining Board just for the fun of the thing. Now, gentlemen, it is remarkable that a man would pay twenty-five dollars, and give three days' hard work, for the sake of having fun with the Association. I do not think the boards are too strict. I am sure if they err at all they err on the side of leniency. There are too many men creeping into dentistry who do not belong there. I believe the boards are trying to be fair in their examinations; they do not put any catch questions or examine upon anything that does not pertain to practical dentistry, and I do not believe that we can find one man who has passed the boards that are moving in that line, who will say that he has ever had anything but a fair and square examination; that if he failed he failed on points that he ought not to have failed on, and was glad he was compelled to acquire the knowledge before he passed. We have had a man who had been before the board eight or nine times before he could pass, afterwards come before us and thank the board for making him obtain the knowledge necessary to make him a professional man.

*Dr. Pruden.*—I hardly think that placing a student under probation is practical or feasible, for this reason, that dentists of to-day are very much opposed to taking students. I have taken two, and do not wish to take another. I was myself the innocent cause of making my preceptor come to the same conclusion. I think if you depend on filling the ranks from students thus admitted the profession would suffer by depletion.

*Dr. Eaton.*—May I answer that question, Mr. President. The time was when a young man wanted to study dentistry he went to a reputable dentist and he paid for the knowledge that he received. Now they expect pay. There is the mistake. If the colleges compelled them to serve six months in the office of some reputable dentist before the college would accept them, the private preceptor with whom they serve this time would be the judge, after six months' trial, of whether they were fit to take a college course and become dentists or not.

*Dr. Graft.*—Mr. President, I suppose I may be said to have both practical experience and theory. I had ten or twelve years' practical experience before I went to college, and since I left college I have been practising dentistry. In my opinion the proper way would be to require all prospective students to have some practical knowledge before they enter college. If a man had to serve six months with a good preceptor I think it would be the best thing that could happen to him. At the end of that time he or his preceptor could tell whether he would ever make a good professional man or not. I believe a law to that effect would be a very good thing for the colleges to take up.

*Dr. Marsh.*—My practical experience has been somewhat different from the usual order. My probation came after I graduated. I had no preceptor. I went through college, and flattered myself that I passed very creditable theoretical examinations. After I left college I soon realized that I did not know everything in dentistry. I was fortunate in obtaining a preceptor in a very excellent dentist. When I finished with the college education which they gave me, it was my good fortune to be a lecturer on oral surgery for a number of years in a Western college, and I found that theory in college was an excellent thing, and I also found that I did not know everything; I had to study almost as hard to keep up as I did when I was in college. Those experiences have taught me that we do not learn everything in college. As one speaker said, young men go to college with the idea that they have nothing more to learn when they graduate. On the contrary, the college teaching is only the beginning of knowledge; it is only a good start in the right direction, and it depends upon their subsequent work and study whether or not they make a success in dentistry, provided they have the necessary natural ability.

*Dr. Twilley.*—Mr. President and gentlemen, really I do not think there is anything left for me to say. It has all been said. I will say this much for myself: I came before you this evening with some little fear and trembling; I had been with the members before in other quarters, and I expected really a good old fight; but I do not feel very much flattened out by the criticisms and remarks made on my paper. I think that I did not make myself quite clear to several of the gentlemen who have spoken; for in the discussion they have really given me credit for thoughts in the paper which I did not have at all. I most undoubtedly hold that the college does not give the finish to the education of its graduates. It is only a primary school. Unfortunately, a great many young men come out of college with the impression that they are the equals of any man in the profession, and the superiors of many. That is a misfortune.

The colleges are raising the standard of graduation to some extent, as some one has said, theoretically, but you must look to the State boards if they are raising it too rapidly. They certainly wish that the graduates shall be thoroughly equipped, evenly equipped, if possible, and they do all in their power to make them so; but it is very difficult, indeed, to have a body of men go out from school all equally qualified to practise dentistry. Go into the artist's studio, and you will see a number of students all using the same paints and the same brushes, but, oh, the difference in the pictures! So it is with the men who walk out of college as new dentists,—there is a great difference in the pictures.

On motion, the subject was passed.

*The President.*—Incidents of office practice are now in order.

*Dr. Meeker.*—Mr. President and gentlemen, I am at present greatly interested in the subject of cataphoresis for the purpose of bleaching teeth and obtunding sensitive dentine, and I know other members here are in the same condition. In the past year I have used the Edison machine; now I use the Wheeler volt-selector. I have had a great deal of success with it, and I must say that the best success I have had in bleaching teeth was in a case that I treated about ten days ago,—a tooth with two gold fillings in it. I had treated it for three months for abscess, had succeeded in getting it in good normal condition, and I bleached it nicely in thirteen minutes with eleven volts measurement of the Wheeler machine.

I think that is very good. I have not been able heretofore, with the use of pyrozone, hot air or dry air, to thoroughly bleach a tooth with a gold filling in it, but with this volt-selector I have been able to drive the pyrozone through the tubuli of the tooth right up to the gold filling. I have been using a number of medicaments in obtunding sensitive dentine: I have used cocaine, I have used atropine a little during the past week, but my best success has been with Parke Davis & Co.'s hypodermic tablets. This formula is hydrochlorate of cocaine, morphine, and sulphate of atropine. In the use of these tablets I take six drops of salt water, and apply the positive pole to the dentine of the tooth. The shortest time consumed in rendering anæsthetic the sensitive dentine of a tooth has been thirteen minutes, with five volts. To-day I tried McKesson & Robbins's new guaia-cocaine,—the aqueous solution,—having a rubber dam on. It was a cervical cavity, and it took me fifteen minutes. It may be that the action of the current was not strong enough, but after applying it that time the tooth was anæsthetized so that I could cut without any pain whatever to the patient. To-day was the first time I had a chance to use it,—I have had the appliance only a week. I have used hydrochlorate of cocaine electrically, and it has done very good work, but this little tablet of Parke Davis & Co. has so far seemed to be the best. I am not prepared yet to say positively it is the best. I think if those who are using this machine will try these different remedies, and keep account of the results obtained in each case, we will finally come to a remedy that will give us the desired effect in a very short time, and in the end time will be saved in filling the teeth.

*Dr. Eaton.*—May I ask Dr. Meeker in how many cases he obtained total anæsthesia?

*Dr. Meeker.*—I have had no case in which there has been total anæsthesia. I have had cases where I used the agent for five minutes, began to cut, and then found sensitiveness; then I applied it for five minutes more, and so was able to prepare the cavity. When the patient complained of any pain or excessive sensitiveness, I would stop and turn on the current again. I have heard a number of gentlemen in the profession say they have used as high as forty volts on a tooth. I have not thus far used over fifteen volts.

*Dr. Eaton.*—Mr. President, I had a case of two lower twelfth-year molars with crown cavities. I placed the negative pole in the hand of the young man, and ran the current up to forty volts without giving any sensation. Then I placed the negative pole on the side of the face and ran the current up to forty volts without having any sensation whatever; and I had the same result with similar teeth on the other side.

*Dr. Osmun.*—Mr. President, it is unfortunate at the present day to have heard of these new things and meddled with them, because you are called on before you have had much experience to explain the results obtained. I have what is called the Van Woert cataphoresis machine, made by Wade & Bartlett, of New York. I am not wildly enthusiastic over it at the present time. I think it has some merit; I don't think it is a panacea for all the ills that patients have had to suffer in having teeth filled. I have had a very peculiar experience in the use of it. Sometimes the result has been satisfactory, sometimes it has been unsatisfactory. I am not disposed, with my present knowledge, to condemn the machine as much as I am to condemn the operator. I think the ignorance of the operator has more to do with the failures than has the machine, and that seemed to be the opinion of my friend Dr. Brown this morning; he assured me it was simply want of a little more practice and knowledge on my part with reference to manipulating the machine, and with that I would have no further difficulty. He said he had the same difficulty in the beginning, but he had overcome it. My theory for several years has been, in reference to obtunding sensitive dentine, that the best method is to have clean, sharp burs, steady hands, an engine revolving at a moderate rate of speed, but running steadily, telling the patient you are going to hurt him a little, and then doing the work deftly and quickly. Up to the present time, I have not found anything that I would trade off for my sharp, up-to-date instruments, deftly handled.

*Dr. Gregory.*—When Dr. Gillett was before the State Society with his machine demonstrating the use of the electric current in the introduction of cocaine, I became very much impressed in its favor. I had no volt-selector, but I did have a four-cell battery. I happened to have a patient, a boy twelve years of age, for whom I had to place a filling in each of the superior central incisors. The pulp was very nearly exposed, and he was an extremely nervous boy; I thought I would try Dr. Gillett's method in that case. I used a saturated solution of cocaine, applying one of the electrodes, the positive, to the tooth, and had the boy grasp the other electrode in his hand. I thought about one volt of current was severe enough. Not having a rheostat, I hardly knew how to manage, and I took the negative electrode and applied it to his ear, and in that way controlled the current. After applying the current for seven minutes I was able to make the necessary grooves and build down the teeth with gold. The boy did not complain a particle. That has been a very great argument with me in favor of cataphoresis.

*The President.*—I will call on Mr. Wheeler to tell us something about cataphoresis.

*Mr. Wheeler.*—Mr. President and gentlemen, I don't know that I can add anything to what has been said on this subject here to-night, with the exception of a little on the subject of electricity and rheostats, and the reasons why you cannot expect to have successful cataphoric action in dental practice without pain when using a rheostat as the means of controlling the current.

A rheostat is a resistance, an obstruction, to the flow of electricity. It is placed in circuit between the source of current-supply and the patient. Increasing the value of this obstruction decreases the current, and *vice versa.*

Now, in actual work on tooth-structure you have other ever-varying obstructions or resistances to the flow of electricity that are entirely beyond your control. The most important of these is the ever-varying resistance of the tooth itself, its dentine, and pulp. Then you have the ever-varying resistance of the solution intended to be driven in, together with the varying joint resistance of both dentine and solution as the solution is carried into the dentine. These variations entirely preclude the use of rheostats, or so-called current-controllers, for painless, rapid, and successful cataphoric work, by reason of the fluctuation of electro-motive force or potential at the tooth terminals, which, according to well-defined laws, must follow the changes in value of resistance. As the potential increases at the tooth-terminals in proportion to increase of resistance of the tooth, so the pain increases; and if by chance, as often happens, connection should be broken, a severe shock to the patient must ensue if sufficient current is passing to accomplish the purpose within a reasonable time.

The above facts will account for a great deal of the pain talked of by those who use that class of apparatus.

About the length of application required I find great diversity of opinion. I have had practitioners tell me that they have successfully obtunded the parts to be operated on in a minute and a half or two minutes. I myself have never produced complete anæsthesia in less than five minutes; in a great many cases it has been ten or twelve minutes, and I have been as long as twenty minutes. Care is required in placing the poles of the battery. Be sure to lead the positive pole to the tooth of the patient. It is so easy to change about, and lead the negative pole to the tooth without knowing it, and then you wonder why you do not get anæsthesia, and proceed to condemn the apparatus and cataphoresis generally. Of course, the negative pole will not produce anæsthesia ; the medicament is driven in only from the positive pole, and the higher the voltage you can attain the quicker the operation. A tooth requires more time to anæsthetize than the soft tissue, partly on account of its comparative density of structure. The resistance of a tooth is much more than that of other tissues. And this resistance varies a great deal. In young persons' teeth the resistance is much less than it is in those of elderly people. This difference is ascribed to the fact that the dentine in the teeth of young persons is looser in structure than it is in those of older persons. I find that the resistance in one tooth will sometimes vary as much as thirty or forty thousand ohms during one operation. It is necessary to keep a steady voltage; it will not do to let it race up and down, as by rheostat control.

*Dr. Meeker.*—Mr. President, I would like to ask Mr. Wheeler what is the best medicament for accomplishing anæsthesia that Dr. Morton uses on the teeth, the hard and soft tissues?

*Mr. Wheeler.*—Guaia-cocaine has been, in my experience, the best,—and I think I can speak for Professor Morton also,—because it does the work quicker than cocaine alone would, and the effect that guaiacol has with cocaine is to localize the anæsthesia. It will not allow the cocaine to be given off in the circulation as quickly as it is if a simple aqueous solution be used. I understand that there is another medicament that Professor Morton is now at work on, which is to be another step in advance in this direction.

*The President.*—Will Dr. Evans favor us with some light on this subject?

*Dr. W. J. Evans.*—I think I can promise you, Mr. President, that within a short time there will be a new salt developed from this union of guaiacol and cocaine which will be presented to you, with the result of enabling you to do things which will be worthy to be written about. It would only be natural that a dentist should be the one to do things before he writes about them rather than write about them before he does them; it would be in line with everything that has been said here to-night. I can assure you, my friends, that it gives me great pleasure to announce to you that, through the investigations of Professor Prescott, of Ann Arbor, you will be presented with an entirely new salt, but it is not yet ready. The solution which Dr. Meeker spoke of to-night, the aqueous solution of guaia-cocaine, contains twenty per cent. of the product resulting from the union, consequent on the affinity which cocaine has for guaiacol; and it will be a result of that which will give you something which Professor Morton thinks will be superior to cocaine, something which you can use without any of the caustic or toxic effects, or bad odor, or any of the objections that you have found with guaiacol.

I was very much interested to-night in the discussion about the qualification of dentists. It seems to me that the qualification to do good work fills a place of very great importance the moment you take up the study of cataphoresis. It is entirely futile and a waste of time to make arguments against cataphoresis, because cataphoresis is a physical fact. It has been demonstrated that the electrical current will make certain fluids move from the positive pole, without resistance, towards the negative pole, and no more argument is necessary on that subject. The means by which you are able to produce that result offer a very large field for investigation; it will be a very interesting field, one in which the practical dentist, with his mechanical skill, will probably be able in the end to do things worthy to be written. Until we hear from Professor Prescott just what is the precise nature of the discovery that has been made, I am not in a position to make any further announcement, but I can promise you something quite important, which will verify what has been published by a Frenchman in this connection. We hope to be able to retain in guaia-cocaine the anæsthetic properties of guaiacol without its objection, added to the full force of cocaine.

*Dr. Meeker.*—Mr. President, if, as Dr. Evans says, we are on the eve of the discovery of a new anæsthetic which will possess the properties of cocaine without its toxic effect, then dentistry in the future is going to be easy work, and the up-to-date dentist will do that work, and not the dental associations that inject hypodermic solutions into the gums.

*Dr. Eaton.*—Mr. President, it is very much to be desired that we shall be able to use cataphoresis in teeth that have already one filling in them. I have found much trouble in such cases. It causes a great deal of pain, and I am going to ask Mr. Wheeler if he will give us the benefit of any discovery he has made in regard to leaving that condition of pain.

*Mr. Wheeler.*—The one thing to do in such cases is to thoroughly insulate the filling that is in the tooth with sandarac varnish. Dr. Gillett uses Gilbert's temporary stopping. I think a shellac varnish would be a better insulator. If you thoroughly insulate the filling in the tooth you will have no trouble with it.

*Dr. Eaton.*—The question I want to get at is this: the electricity having some affinity or finding less resistance in a filling, after it passes through a portion of the tooth, the fluid having been forced through a portion of the dentine and coming in contact with the filling, somehow causes pain near the filling. The temperature of the filling is raised, I believe, and I think that produces pain; is that so?

*Mr. Wheeler.*—No; the effect of the metal in the tooth would have a tendency to lower the temperature in the tooth.

*Dr. Eaton.*—Why do we get pain in that condition, where a tooth has a filling in it?

*Mr. Wheeler.*—The only thing that could give pain in that condition, to my mind, would be the incipient electrolysis of the metal in the tooth. There is a tendency of the anæsthetic to be drawn towards the metal in the tooth instead of directly down towards the root-canal. I have noticed that a tooth becomes anæsthetized first in the portion towards the filling. I have also noticed that it takes a little longer to obtund teeth with filling in them, for the reason given. The resistance of a tooth is very great as compared with that of the soft tissue, and in teeth of people of advanced age I have found very high resistance.

*Dr. Meeker.*—That is because the lime-salts are more calcified.

*Mr. Wheeler.*—I suppose so. I know that the older a person is the slower is the cataphoric action.

*Dr. Adelberg.*—Mr. President, my experience with cataphoresis has been of a somewhat varied character. I have had most remarkable success in some cases, and some wonderful failures in others. For instance, I have drilled into a tooth and taken out an amalgam filling, using cataphoresis, in an upper molar, drilling into the nerve-canal and extracting a mummified pulp; and then at other times it produces great pain. The mummified pulp I have here in my pocket. I had a very queer experience in the use of cataphoresis on a lady, a woman weighing two hundred pounds. The peculiar part of it was the effect on her fingers. She wore rings and could not get them off, she was so fat; a few days after the operation she came back to me with blisters on her fingers under the rings. I had used cataphoresis to the fullest extent, pumping it up to forty volts; she did not complain of any pain, although the tears ran out of the corners of her eyes. I was using it on an upper third molar that had two cavities, and I managed to get the sensitiveness down so that I could excavate them. The lady had a highly nervous temperament, her teeth were extremely sensitive, and during the operation she complained of the negative pole hurting her fingers, especially around the rings. I told her to wrap her handkerchief around the rings. I ran the current up to forty volts, and could excavate the tooth, although I did not get full anæsthesia. I thought I would report this case, as I had never heard of a similar experience, and I would like to know if others have had the same result. I am sure the blisters on the fingers must have been caused by the negative pole, because she did not have anything else in her hand.

*Dr. Brown.*—Mr. President, I gave my experience with cataphoresis at the last meeting, and if I were to say anything now it would simply be a restatement; but from my experience I can support Dr. Adelberg in his views. I had a case where there were blisters raised under the rings where the negative electrode was held in the hand. The patient complained of the sensation at the time, but I desired to keep the electrode in her hand; I wanted to see what it would do. I think it would be advisable to have the patient take the negative electrode in the hand that contains no rings on the fingers, and I have adopted that method ever since. The guaia-cocaine salt will certainly be a wonderful thing if it proves as efficacious as it is supposed to be. At our last meeting Mr. Woolf said he would do a little work in this line and report to you. He has, in connection with one of the chief chemists of the New York Board of Health, been working in this direction, and a few days since he sent me certain preparations which they claim will completely revolutionize this whole business of cataphoresis. I cannot tell you what they are, I could not promise their names; but if these new preparations show up as they say they will, I think, after we write the names on the black-board at Asbury Park, we will really have something to publish.

On motion, the subject of Incidents of Office Practice was passed.

*Dr. Meeker.*—Mr. President, I move that a vote of thanks be given to Dr. Twilley; and I also move that Dr. Twilley be made an honorary member of this Association. He has been with us a number of times at our meetings and has shown some interest in the Association both at Asbury Park and Newark.

Dr. Meeker's motions were carried.

Adjourned to the third Monday in September.

